# Biofilms in Endodontics—Current Status and Future Directions

**DOI:** 10.3390/ijms18081748

**Published:** 2017-08-11

**Authors:** Prasanna Neelakantan, Monica Romero, Jorge Vera, Umer Daood, Asad U. Khan, Aixin Yan, Gary Shun Pan Cheung

**Affiliations:** 1 Discipline of Endodontology, Faculty of Dentistry, The University of Hong Kong, Pok Fu Lam, Hong Kong, China; spcheung@hku.hk; 2 Department of Endodontics, Benemerita Universidad Autónoma de Puebla, Puebla 72000, Mexico; moniendo@gmail.com; 3 Department of Postgraduate Endodontics, University of Tlaxcala, Private practice, Puebla 72420, Mexico; jveraro@yahoo.com.mx; 4 Faculty of Dentistry, International Medical University, 126, Jln Jalil Perkasa 19, Bukit Jalil, 57000 Bukit Jalil, Malaysia; udaood@hotmail.com; 5 Medical Microbiology and Molecular Biology Laboratory, Interdisciplinary Biotechnology Unit, Aligarh Muslim University, Aligarh, Uttar Pradesh 202001, India; asad.k@rediffmail.com; 6 School of Biological Sciences, The University of Hong Kong, Pok Fu Lam, Hong Kong, China; ayan8@hku.hk

**Keywords:** bacteria, disinfection, extracellular polysaccharide, irrigation, root canal, review

## Abstract

Microbiota are found in highly organized and complex entities, known as biofilms, the characteristics of which are fundamentally different from microbes in planktonic suspensions. Root canal infections are biofilm mediated. The complexity and variability of the root canal system, together with the multi-species nature of biofilms, make disinfection of this system extremely challenging. Microbial persistence appears to be the most important factor for failure of root canal treatment and this could further have an impact on pain and quality of life. Biofilm removal is accomplished by a chemo-mechanical process, using specific instruments and disinfecting chemicals in the form of irrigants and/or intracanal medicaments. Endodontic research has focused on the characterization of root canal biofilms and the clinical methods to disrupt the biofilms in addition to achieving microbial killing. In this narrative review, we discuss the role of microbial biofilms in endodontics and review the literature on the role of root canal disinfectants and disinfectant-activating methods on biofilm removal.

## 1. Introduction

A biofilm is a highly organized structure consisting of bacterial cells enclosed in a self-produced extracellular polymeric matrix attached on a surface. Biofilms may also be considered as a layer of condensation of microbiota or a microbial-derived community consisting of cells that are irreversibly attached to a substratum or interface or to each other, and embedded in a matrix of extra-cellular polysaccharides in addition to extracellular DNA (eDNA) and extracellular proteins [1,2]. In general, the exact composition varies with the microorganisms and nutrients available. The organisms in the biofilms exhibit an altered phenotype with respect to growth rate and gene transcription [3,4].

Sessile bacterial cells (biofilm state) differ greatly from the free-floating bacterial cells (planktonic state). The physiological properties of bacteria in biofilms are different compared to the same bacterium in a culture media, partly because microorganisms in biofilms are protected from environmental stresses by their matrix [5]. Microbes within the biofilm can be about 1000-fold more resistant towards antimicrobial agents and host defense mechanisms than their planktonic counterparts [6,7]. Bacterial cells grow more slowly in biofilms than in their planktonic state and therefore, take up antimicrobial agents more slowly [8]. The biofilm also provides a ready environment for mutation accumulation in microbial cells, further promoting their survival and persistence.

The process of root canal treatment involves enlarging the root canal with instruments and cleaning the space using chemical disinfectants to (i) remove remnant vital or necrotic tissues; (ii) kill the microbiota within the root canal system, including disruption of the microbial biofilm and (iii) remove the accumulated hard tissue debris that is formed during root canal instrumentation [9,10]. In general, the aim of any disinfection strategy in healthcare is to reduce the bacterial load to a subcritical level so that the patient’s immune response will allow healing. Root canal treatment is no different where root canal disinfection is considered the pivot of this therapy [11,12]. Interestingly, as elsewhere in the body, the critical threshold that is compatible to health is unknown and hence endodontic research has focused on developing methods that can completely remove the bacterial biofilm, which is the ideal goal.

Microbial biofilms in the root canal are highly resistant to disinfecting agents used in endodontic treatment. The complex and unpredictable nature of root canal anatomy and the multi-species biofilms amplify the difficulty in eradication of the microbial biomasses from there [13,14]. The objective of this review is to discuss the microbiological aspects of root canal biofilms, clinical antibiofilm strategies and research methods to study biofilms.

## 2. Microbiology of Root Canal Infections

The bacterial microflora of the root canal is initially dominated by aerobes and facultative anaerobes [15,16]. As disease progresses, the ecology within the root canal system changes. Such changes maybe related to the oxygen tension when root canals are opened up during treatment, use of root canal irrigating agents and changes in the canal pH due to various materials introduced into the root canal. This results in phenotypic changes driven by genetic population shift [17,18,19]. Endodontic infection may be primary or secondary. In general, primary infection involves pulp inflammation and root canal infection following invasion by microbes or microbial by-products, eventually resulting in inflammation of the supporting tissues i.e., apical periodontitis. Secondary infection (or post-treatment infection) occurs either as reinfection (acquired or emergent), remnant (persistent) infection or recurrent infection (re-developed in teeth after apparent healing) in teeth that have been previously root canal treated [11]. Primary endodontic infections are polymicrobial [19,20]. They are predominantly *Bacteroides*, *Prophyromonas*, *Prevotella*, *Fusobacterium*, *Treponema*, *Peptostreptococcos*, *Eubacterium*, and *Camphylobacter* species.

It is believed that persistence of microorganisms within the root canal system after treatment is the major cause of treatment failure [21]. The ratios of the microbes in primary infections could be different after root canal treatment, as well as a shift in species propagation and quantity. The microbial flora found in secondary infections, typically, are able to survive harsh conditions such as a wide pH range and nutrient-limited conditions. There is a definite contrast in the microbial phenotypes in primary infections as compared to secondary infection, with the latter being predominated by gram-positive bacteria [22,23,24]. Studies have shown the prevalence of certain species in teeth with post-treatment infection, such as *Enterococci*, *Streptococci*, *Lactobacilli*, *Actinomyces* and fungi (such as *Candida*). In particular, a high proportion of *Enterococcus fecalis* in cases with persistent apical periodontitis was noted [25,26].

Mixed infections are more common than single-organism isolates. Also, the wide variety of organisms found in root canals can be partially related to the principal interests and culture techniques of different investigators. Isolates from the exposed pulp are similar to the oral flora in which gram-positive cocci predominate, and approximately 25% of the isolates are anaerobes. Organisms associated with flare ups (which are emergency conditions characterized by pain and/or swelling) seem to share a similar composition as those from asymptomatic root canals [27,28]. These are usually phenotypic changes due to ecological shifts. Organisms cultured from infected canals elaborate a variety of invasive enzymes, but it is unclear if it can be equated with pathogenicity [29,30,31].

## 3. Characteristics of a Biofilm

Bacteria in the state of a biofilm are able to survive tough growth and environmental conditions, which, in part, is due to the protection offered by the extracellular matrix of the biofilms. This structure enables trapping of nutrients and allows metabolic cooperation among various resident bacteria of the same or different species. The organized internal compartmentalization in a biofilm allows bacteria of different growth requirements to survive in their own microenvironments.

### 3.1. Protection of Biofilm Bacteria from Environmental Threats

Bacteria are capable of producing cell surface structures (e.g., capsule) or extracellular secretions (e.g., extracellular polysaccharide). The extracellular polysaccharide (EPS) can offer protection to all resident bacteria from various environmental stresses such as pH shifts, osmotic shock, UV radiation and desiccation [32,33]. It also alleviates the effect of any harmful substances that have to diffuse through the EPS matrix before reaching the microorganisms.

### 3.2. Enhanced Tolerance to Antimicrobials

Long term use of drugs leads to the development of resistance among microorganisms due to altered gene expression and transfer of resistance genes, rendering the antimicrobial agent ineffective [32,34]. EPS matrix in bacterial cells acts as a barrier, trapping extracellular enzymes such as β-lactamase and thus inactivates β-lactam antibiotics. Due to the depletion of nutrients, bacteria are forced into a dormant state and thereby protected from being killed readily [35].

Another mechanism responsible for antimicrobial tolerance is the selective location of anaerobic niches deep within the biofilm community, as oxygen can be completely depleted by bacteria at the biofilm surface [36]. A sub-population known as persisters is often found within the biofilm community. These persisters can belong to any bacterial type and are in a highly resistant phenotypic state that is resistant to killing by many antimicrobial agents [37,38].

### 3.3. Quorum Sensing

Quorum sensing is a bacterial cell-to-cell communication system [39]. Using chemical signaling molecules, bacteria are able to communicate with one another. For example, molecules such as the competence-stimulating peptide (CSP) are important to co-ordinate gene expression. Quorum sensing allows bacteria to monitor the environment for other bacteria and allow alteration of one’s behavior in a population-wide scale. Microorganisms function less effectively as individuals compared to coherent groups [40]. Pooling the activity of a quorum of cells can improve the successful persistence of bacteria.

Quorum sensing is known to be involved in the formation of biofilms and to cope with environmental stresses [41]. The characteristics of a specific strain of microbe determine its ability to co-exist. Such an association has been demonstrated between *E. fecalis*, *Streptococcus gordonii* and *Lactobacillus salivarius* [42]. One reason for this could be the differential starvation endurance of *E. fecalis* in mono-species and multi-species biofilms, depending on which microbe it is in association with. The protease production in biofilm environments appears to have an influence on the ability of co-existence between bacteria, as it is related to the virulence of bacteria [42]. Specific quorum sensing genes have also been implicated in the biofilm forming ability of *E. fecalis* (e.g., *S*-ribosylhomocysteine lyase [luxS]). LuxS encodes autoinducer 2 which is considered a cross-species signaling molecule [43]. It is surprising however, that quorum sensing and its inhibition have not been well studied within the root canal system. Future research should focus on using quorum-sensing inhibitors in root canal antibiofilm strategies.

**Figure 1:**
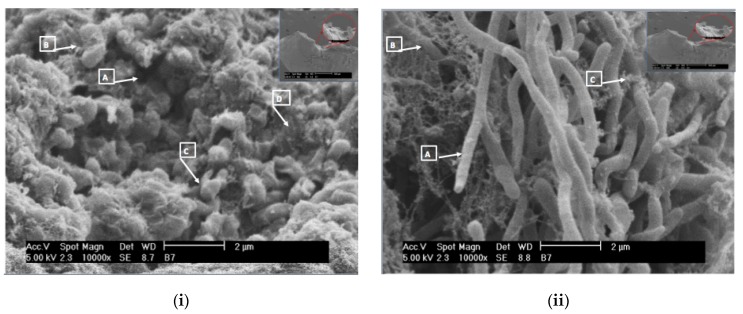
Scanning electron microscopic images of the apical of the apical foramen of a tooth showing a mature biofilm of (**i**) *Leuconostoc* spp. (10,000×). A multilayered polymeric matrix is evident. Also noticed are communicating channels in the biofilm (A), extracellular fibers secreted by bacterial cells (B), metabolically active cells characterized by cell division (C) and compact extracellular polymers (D); (**ii**) *Biofidobacterium* spp. in a maturation state (10,000×). Secretion of polymers in granular form by the bacterial cells (A), microfilaments of extracellular polymers surrounding the rod shape of the microorganism (B) and extracellular polymers in the maturation state (C) can be seen (Courtesy: Dr. Ana Maria González Amaro, Maria Verónica Méndez González, UASLP Mexico).

**Figure 2:**
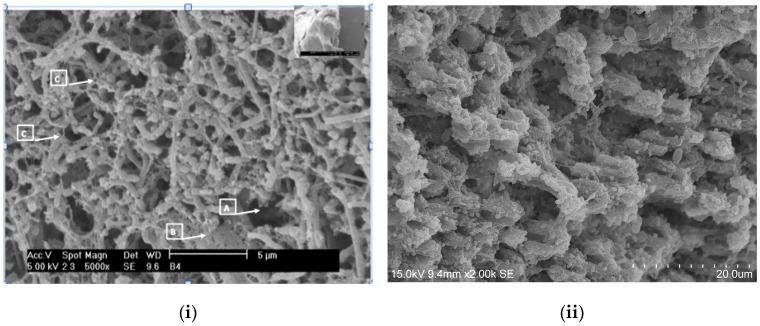
Scanning electron microscopic images of (**i**) *Clostridium botulinum* and *Streptococcus anginosus* (5000×). Communication channels inside the biofilm (A), fusion of extracellular fibers in laminar shape (B) and the presence of numerous polymeric fibers secreted by rods and cocci forming a crisscross pattern (C) are evident. (Courtesy: Dr. Ana Maria González Amaro, Maria Verónica Méndez González, UASLP Mexico); (**ii**) 3 weeks biofilm of *Enterococcus fecalis* on dentin (2000×). The dense EPS matrix is visible and the challenge in eradicating biofilms of this microbe is, in part, attributed to the ability of medicaments to penetrate this matrix. This is the most commonly implicated bacterium in root canal treatment failure.

**Figure 3:**
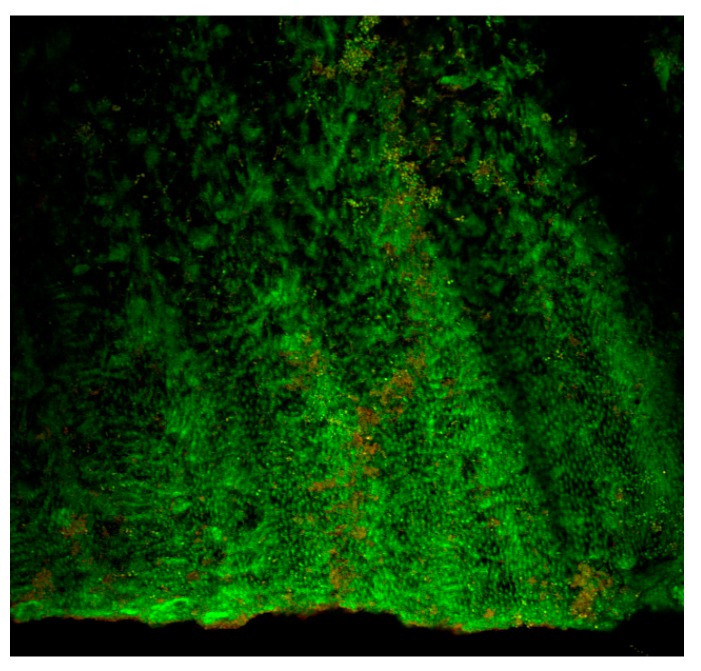
Confocal laser scanning microscopic (CLSM) image (single slice at 20× magnification) of a 1 week biofilm of Enterococcus *fecalis* within the root canal. Dense aggregates of bacteria are seen within the dentinal tubules. The advantage of CLSM imaging is to detect the presence of apparently dead (red) and apparently live (green) bacteria. It is also possible to perform 3 dimensional reconstruction of Z-stack images to study biofilm architecture.

Figure 1 and Figure 2 show some multi-species biofilms from the apical foramen of root canals demonstrating communicating channels, fusion of extracellular fibers, compact extracellular polymers and their network. Figure 3 shows a mono-species biofilm of *Enterococcus fecalis*, with its dense EPS matrix.

## 4. Methods of Studying Biofilms in Endodontics—Concepts from the Non-Endodontic World

Contemporary microbiological research has helped us develop an understanding that interactions between microorganisms are complex and play an important role in pathogenesis, either by synergistic or antagonistic mechanisms. This understanding has led to increased interest in studying the mechanisms involved and to a shift away from being monomicrobial to polymicrobial nature, when studying root canal biofilms in vitro [44,45]. Recent studies have focused on multi-species bacterial biofilms as a more realistic reflection of what happens in the real in vivo situation in the root canal system. Endodontic infection demonstrates significant microbial diversity, and the properties of such complex biofilms cannot be observed in studies with one species alone [4,46,47,48].

It is known that the functional properties of biofilms are intimately related to their 3-dimensional structure. It is necessary to understand and to study this architecture, not only to observe them on a cellular scale, but also in relation to the extracellular matrix, which creates such a special ecosystem. To achieve this objective, it is necessary to use techniques that allow observation of reconstructions of the communities [49].

Appreciation of bacteria living in multi-species biofilms rather than in mono-species communities, or in a free-living state, has changed the way bacteria are studied in the laboratory. Models are being created to replicate biofilm environments [50,51], thereby entering times of re-learning bacterial behavior as they live and survive in biofilms. In the same way, meta-genomic studies have increased knowledge of the complexity of microbial relations within the biofilm population [31,52].

Nowadays, biofilms are studied in two ways: in one, the consortia of microorganisms as a single unit and, in the second, by studying the effects and relationships between one species and others [44]. Because of advances in technology and computational biology, it is possible to study gene and protein expression in such communities, thus revealing the role that each species has in that specific community [53]. Pragmatically speaking, identification of the exact nature of an intracanal biofilm is a real challenge, because few techniques are capable of re-creating both the extracellular matrix as well as the microorganisms in that biofilm.

There are numerous advantages of using an in vitro biofilm model, which include ease of modification if necessary, control of variables, low cost and ease of replication. They are also very useful in answering some initial but fundamental questions, providing preliminary data, which is essential for future confirmation by in vivo testing.

### 4.1. Microtiter Plate-Based Systems

These are useful and consistently used biofilm model systems. The system is closed; therefore, there is no flow in or out of the reactor during experimentation. Consequently, the environment in the experimental model changes, for example, with regard to the availability of nutrients and molecules [54]. The microtiter plate-based system is used to perform different tests at the same time, which may be ideal for rapid screening of methods for biofilm disinfection and removal [55]. Biofilm quantification with microtiter plates may be categorized into biomass assays, viability assays and matrix quantification assays [56].

Biofilm analysis on microtiter-plate based systems may be performed using crystal violet, nucleic acid stains such as Styo9, non-fluorescent fluorescein diacetate, tetrazolium salts such as XTT, resazurin or dimethyl methylene blue. These methods show differential results for fungal and bacterial biofilms. Furthermore, it has been suggested that the Styo9 assay should not be used for CFU measurements in biofilms and that the crystal violet assay was non-repeatable for *Pseudomonas aeruginosa* biofilms (56). A limitation of the Styo9 assay is that it depends on microbial cell wall integrity, and thus can be misrepresented by microbes that may be dead, yet have an intact cell wall. CFU counts only reproducing cells, and can over-quantify killed cells based on reduction of metabolic activity (chemostatic treatments would be considered killed). Since these test methods measure different analytics to describe viability, it is quite possible the results do not match. Hence, it may be preferable to perform tests such as FDA or resazurin for quantification of biofilms with differentiation between dead and live cells [56].

### 4.2. Flow Displacement Biofilm Model Systems

In contrast to the microtiter plate-based system, the flow displacement system is open. In this system, the growth medium containing the nutrients needed for growth is added at a constant rate, with the waste products and toxins from the biofilm removed simultaneously [57]. The concept of flow displacement is based on the premise that an initial film of macromolecular components needs to form on a surface to allow microbial adhesion [57]. The fluid flow, in an optimal manner ensures adhesion of microbial cells to a substrate, which is a characteristic property of any biofilm.

It has been suggested that experiments using parallel plate flow chamber performed with controlled hydrodynamic conditions would offer ideal flow rates for microbial adhesion and reduces technical variables [58]. This also helps in repeatability of the study designs in different laboratories.

### 4.3. Modified Robbins Device

The modified Robbins device is a device in which there is a continuous formation of biofilm which is exposed to fluid flow [59]. This device may employ silicone or hydroxyapatite discs as the substrate for biofilm growth, with or without addition of agents that support or inhibit microbial growth. The main advantage of this system is that it allows evaluation of more than one antibiofilm agent in the same experiment [60]. This device can also be modified and used in conjunction with flow devices.

### 4.4. Microfluidic Device

This is becoming a popular method of studying biofilms, because of the possibility of forming a biofilm under conditions similar to that physiologically, for example, cell-to-fluid volume ratios and flow velocities. Also, since the chamber is small, it allows for a single cell resolution analysis of the biofilm under tightly controlled conditions [61]. In essence, this device allows the analysis of chemical assays using small quantities of liquids on a small chip. Such an approach could be very useful in techniques such as polymerase chain reaction, protein analysis and DNA sequencing [62,63]. However, there are challenges in this approach in terms of analysis of biofilms, with specific reference to quantification using methods such as fluorescent staining [64]. An interesting development in this aspect was the application of a modified confocal reflection microscopic approach, termed the continuous optimizing confocal reflection microscopy, to quantitatively study the biovolume of biofilms [64].

In essence, various techniques and technologies have helped to increase our understanding of biofilm physiology and the behavior of bacteria living there. However, these experiments are performed on in vitro biofilm models that may not accurately represent or mimic in vivo biofilm behavior or they simply under-represent it. Another important issue is that in vitro models lack the reaction or challenge imposed on a biofilm in vivo due to the host immune response, therefore limiting the knowledge of how this very important aspect would add to the survival and/or elimination of a biofilm.

### 4.5. Confocal Laser Scanning Microscopy

In recent years, confocal laser scanning microscopy has emerged as a very good method to study biofilms structure [65], because it allows non-destructive investigation of these ecosystems and the hydrated spatial arrangement at cellular scale. The use of fluorescent markers allows targeting of particular cells or, even certain components of the extracellular matrix.

The use of specific stains [e.g., live/dead stains] allows differentiation of live and dead bacteria often times demonstrated by green or red fluorescent signals [66,67] (Figure 3). However, this may be controversial as the processing of a specimen may result in bacterial death and hence falsely reported as efficacy of a treatment approach. Nevertheless, this technique allows us to have a fair idea of the effectiveness of irrigating solutions and techniques in bringing about disruption of the biofilm structure using a three-dimensional reconstruction of the biomass as to study biofilm architecture. Limitations are the same as those for light microscopy, including the fact that it is not able to visualize the cellular ultrastructure, which requires higher-resolution imaging.

### 4.6. Fluorescent Microscopic Techniques with Super Resolution

New microscopic techniques such as STED (Stimulated Emission Depletion), PALM (Photo-Activated Localization Microscopy) and SIM (Structured-Illumination Microscopy) are able to eliminate some of the limitations in terms of resolution [68]. However, for this purpose, the biofilm must be labeled with fluorescing dyes. Hence, it is essential to identify specific markers for the biofilm components that are to be imaged and analyzed.

### 4.7. Scanning Electron Microscopy (SEM)

The use of SEM overcomes some of the limitations related to the use of fluorescent dyes. This technique allows scanning of microbial ecosystems to obtain qualitative information as well as detailed analysis of morphological structures, such as the cellular surface of one bacterium or identification of damage to the cell membrane. It also allows analysis of cell-to-cell interactions by detecting structural changes in the ecosystems [69,70]. However, it must be appreciated that the sample preparation for this kind of microscopy usually involved high vacuum conditions which results in distortion of the extracellular polymeric matrix [71]. Low vacuum methods, such as environmental SEM, may be more useful in this regard.

## 5. Removal of Endodontic Biofilms during Root Canal Treatment

**Figure 4:**
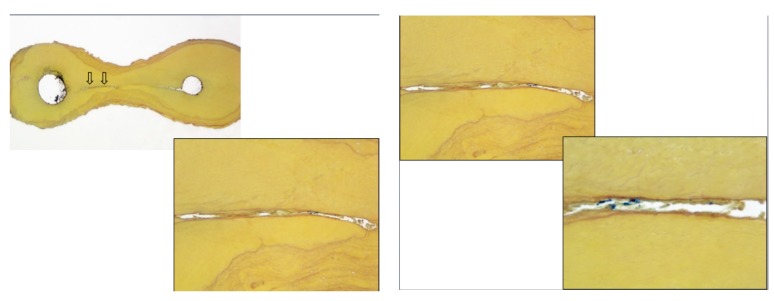
Histological section of the isthmus area between two canals in a mandibular molar, stained by Taylor modified Brown and Brenn stain (16× and 100×) showing the presence of numerous bacterial masses with tissue. A higher magnification (100× and 400×) reveals the presence of residual bacteria and debris in the communications between canals after cleaning and instrumentation of root canal systems. This is the existing challenge in root canal treatment (Courtesy: Dr. Domenico Ricucci, Italy).

The objectives of root canal irrigation are to dissolve vital or necrotic pulp tissues, disrupt endodontic biofilms, neutralize endotoxins and remove the smear layer. Antimicrobial activity and biofilm destruction appear to be the most important objectives targeted towards the etiology of pulp and periradicular infections. Figure 4 show the presence of tissue debris within microbial biofilms in the isthmus between canals in a molar tooth.

The challenge in endodontic treatment is for the disinfectants to reach those minute areas and facilitate removal of the inflamed or necrotic tissue within biofilms. This section of the review will focus on reports concerning biofilm destruction by the most commonly used root canal irrigants. In line with the contemporary understanding of endodontic biofilms, only studies that focus on antibiofilm strategies (rather than just antimicrobial activity) are discussed.

### 5.1. Root Canal Irrigants

#### 5.1.1. Proteolytic Irrigants

*Sodium hypochlorite* (NaOCl) is regarded as the most potent disinfectant in endodontics due to its excellent ability to dissolve vital and necrotic tissues, in addition to its antimicrobial activity [10,72,73]. In endodontic therapy, NaOCl is used in concentrations ranging from 0.5 to 6%, all of which demonstrate antibacterial activity [74,75,76]. Studies show that the antimicrobial activity is not concentration dependent, but tissue dissolution and biofilm disruption are concentration dependent [77,78]. The recommended irrigation regimen involves a sequential use of NaOCl and a decalcifying agent. Ozdemir et al., concluded that the combined application of 17% EDTA and 2.5% NaOCl reduces the amount of intracanal biofilm significantly [79]. The effectiveness of sodium hypochlorite may be improved by warming the solution, use of agitation/activation methods, increasing the volume of the irrigant, and lowering the pH of the irrigant solution [13,80,81]. While the role of warm NaOCl is not clear, owing to the fact that extreme temperature is rapidly buffered within the root canal system [82], it could be advantageous to continuously deliver warm NaOCl via new devices based on negative pressure [83]. Alternatively, techniques such as ultrasonic activation also increases the temperature of the irrigating solution and may prove to be beneficial [81]. The endodontic literature is consistent in demonstrating that NaOCl is able to completely disrupt the biofilms within the root canal system. However, Rosen et al., reported a very interesting finding that NaOCl induces a viable but non-culturable state of bacteria in biofilms and that this might contribute to bacterial persistence [84].

#### 5.1.2. Antiseptics

Chlorhexidine (CHX) gluconate with a very broad antimicrobial spectrum is used as an oral antiseptic mouthwash for plaque control and as an irrigant for periodontal therapy and infected root canals [72,85]. It has a lower grade of toxicity compared to sodium hypochlorite and sustained action i.e., substantivity. A concentration of 2% is recommended as a root canal irrigant [10,86]. Arias-Moliz et al., showed that alternating the application of CHX and cetrimide resulted in a higher percentage reduction of *Enterococcus fecalis* compared to the combined use of these 2 agents [87]. *Cetrimide* facilitates the destruction of EPS matrix allowing CHX to act more directly on *Enterococcus fecalis* thus resulting in a greater bactericidal potential [88].

In two different studies, Baca et al., concluded that the combination of 2% CHX and 0.2% cetrimide as a final irrigating solution showed maximum residual and antimicrobial activity on *Enterococcus fecalis* biofilm [88,89]. CHX Plus, which is a combination of chlorhexidine gluconate with surface modifiers, showed higher levels of bactericidal activity compared to CHX alone [90]. Mechanical agitation of CHX has been proven to promote its antimicrobial effectiveness. Comparing CHX and NaOCl, it is now known that although CHX exhibits antibacterial activity but is unable to destroy the biofilm structure [91,92].

Another bisbiguanide, Alexidine (ALX) was introduced as a root canal irrigant quite recently. Alexidine differs from CHX due to the presence of 2 hydrophobic ethylhexyl groups, which enables rapid antibacterial action. Compared to CHX, ALX has a greater affinity for lipoteicoic acids resulting in an increased permeability into the bacterial membrane [93]. ALX (1%) has been shown to bring about bacterial killing similar to 2% CHX, although both agents do not appear to disrupt the biofilms of *E. fecalis* [94,95]. Octenidine hydrochloride (OCT) is a positively charged bispyridinamine. It acts by binding to the negatively charged bacterial cell envelope resulting in disruption of the functions of bacterial cell membrane [96]. The antimicrobial activity of OCT on *Candida albicans* was studied by Eldeniz and coworkers who reported that OCT was able to totally eliminate all Candida cells when used as a root canal irrigant [97]. Not enough evidence exists at this time evaluating alexidine or octendine on complex biofilms and future research is warranted.

Iodine potassium iodide (IKI) was first used by a French physician, Lugol to treat scrofula in 1829. In 1927, iodine products were initially used as root canal irrigants. IKI has a broad spectrum of antimicrobial action and is effective against enteric bacteria, enteric virus, and protozoan cysts. Due to its lack of tissue dissolving capabilities, it has been suggested to use IKI after canal instrumentation and irrigation with sodium hypochlorite [98]. Wang et al., found that the addition of a detergent to IKI increases the antibacterial effects against *Enterococcus fecalis* in the dentinal tubules [99]. However, the literature lacks evidence on the effectiveness of this compound on the biofilm structure itself.

#### 5.1.3. Demineralizing Agents

Ethylenediaminetetraacetic acid (EDTA) is a chelating agent recommended as an adjuvant in root canal therapy. Many authors have shown its efficacy for removing the inorganic portion of the smear layer [73]. However, EDTA has little or no antimicrobial activity. Alternating the use of NaOCl and EDTA during root canal treatment appears to be a promising approach to remove the organic and inorganic debris, in addition to disrupting microbial biofilms [81]. Soares et al., studied the effectiveness of chemomechanical preparation with alternating use of sodium hypochlorite and EDTA on an intracanal *E. fecalis* biofilm and found that the alternating use of these 2 agents promoted the elimination of root canal *E. fecalis* biofilm [100]. While EDTA has been shown to be effective against *Candida albicans* [101], its efficacy on *Candida albicans* biofilms or multi-species biofilms has not yet been documented.

Another demineralizing agent, maleic acid has been shown to be effective against *E. fecalis* at a concentration of 0.88% for 30 seconds [102]. It is believed that the cell membrane permeability is altered due to the decrease in the internal pH of the microbial cell, resulting in death of the bacterial cell. However, such an action was not shown against intra-orally formed multispecies biofilms [103]. A 2.25% peracetic acid (PAA) solution was recommended as a final irrigant after the use of sodium hypochlorite during instrumentation [104]. Peracetic acid has been shown to be more effective than chlorhexidine against root canal mono-species *E. fecalis* biofilms [91,103]. One may consider peracetic acid as a single irrigant with two purposes—it is a demineralizing agent with strong antibacterial properties [104].

#### 5.1.4. Combination of Irrigating Solutions

MTAD (BioPure MTAD, Dentsply Sirona Endodontics, York, PA, USA) is a mixture of 3% doxycycline, 4.25% citric acid and 0.5% Tween 80. Prabhakar and coworkers showed complete inhibition of bacterial growth by MTAD in a 3 week old biofilm [105]. In contrast, some studies have concluded that MTAD did not have good antibacterial activity against *E. fecalis* [103,106]. QMiX is a mixture of CHX, EDTA and a detergent. It has been shown to be as effective as NaOCl and superior to CHX against *Enterococcus fecalis* and mixed plaque bacteria in planktonic and biofilm states [107,108].

An interesting concept called continuous chelation involves mixing 5% sodium hypochlorite with 18% etidronic acid to serve as a single proteolytic-antibacterial-demineralising solution [104]. Etidronic acid is a weak chelator and hence, when mixed with NaOCl, can be indicated as an irrigant during the entire instrumentation process. The continuous chelation protocol has been shown to bring about excellent antibiofilm activity against biofilms of *E. fecalis* [81,91].

#### 5.1.5. Natural Agents (Phytotherapeutic or Ethnopharmacological Approaches)

Although several studies have evaluated the antibacterial actions of essential oils, none have tested them on biofilm models. Hence, this category of materials is not discussed. Other phyotherapeutic agents such as Berberine, *Morinda citrifolia* and curcumin have also been evaluated against root canal biofilms. Berberine, an antimicrobial plant alkaloid, when combined with 1% chlorhexidine has antibacterial activity comparable to 5.25% sodium hypochlorite and 2% chlorhexidine [109]. It has been tested on *Candida albicans* biofilms in a non-endodontic model, and appears to show favorable antibiofilm activity when combined with miconazole [110]. 

Curcumin is a naturally occurring yellow compound found in *Curcuma longa* L. which is most commonly known as turmeric. Many studies have shown that curcumin has antimicrobial, anti-inflammatory and anti-oxidant activities. Recent evidence shows that curcumin is an effective photosensitizer and brings about antibiofilm activity and dentinal tubule disinfection similar to sodium hypochlorite [6,111].

#### 5.1.6. Nanoparticles Based Disinfection

In recent years, the use of nanoparticles to disinfect root canals has gained popularity due to their broad spectrum antibacterial activity. Chitosan (CS-np), zinc oxide (ZnO-np) and silver (Ag-np) nanoparticles possess a broad spectrum of antimicrobial activity, caused by altering cell wall permeability resulting in cell death [112,113,114]. This topic has been excellently reviewed by Shrestha and Kishen recently [113].

Rose bengal-functionalized CS-np have been widely studied and appear to be effective against monospecies and multispecies biofilms, even in the presence of tissue inhibitors [115]. When attempting to disinfect the root canal system, the priority is on targeting the microbial biofilm and to render the substrate less or not amenable to microbial adhesion. However, it is also important to protect the substrate dentin from further degradation or, at least, not damaged by the irrigants in terms of their physical and chemical structure [116,117].

Photodynamic therapy (PDT) has been used to disinfect root canals because it has antimicrobial activity and the ability to cross-link collagen with proteins. Rose bengal, a non-toxic dye, becomes cytotoxic when activated with a low-intensity visible light and oxygen, targeting cells or tissues in general as well as the lesion to which it is directed [117,118]. Chitin, obtained from crustaceans, such as crabs and shrimps, is another important polymer. From this, chitosan, which is biodegradable and non-toxic, can be derived and used for biomedical and pharmaceutical applications [113,119]. Chitosan can also be decorated with photosensitizers [120,121] and chitosan conjugated with rose bengal has been reported to enhance the degradation resistance of collagen than rose bengal alone, lacks residual activity, and is stable in the environment [115,118].

Silver nanoparticles sized 10–100 nm was demonstrated to possess powerful antibacterial activity against gram-positive and gram-negative bacteria [114]. Furthermore, mesoporous bioactive calcium silicate nanoparticles and bioactive glass powder loaded with AgNp demonstrated significant reduction in adhesion of *E. fecalis* biofilms and this was further exemplified by ultrasonic activation [122]. These materials appear to be potentially useful intracanal disinfectants and further research is needed on the biofilm disruption by these materials.

Nanoparticles with reactive molecules and nanoscale materials have the potential to combat microorganism resistance, since they have the advantages of very small sizes, a large surface-area-to-mass ratio and very good reactivity [123]. PDT used together with nanoparticles has enhanced the efficacy of treatment against microorganisms like bacteria by improving delivery and reducing activation [124]. However, some limitations exist; they can form some aggregates compromising the area where they are used [125] and, because of the anatomical complexities present in the root canal system, effective delivery to target areas may not be possible. Future research must focus on the delivery of nanoparticles into all corners of the root canal system to enable optimal disinfection.

#### 5.1.7. Miscellaneous Interventions

Enzymatic irrigation was introduced by Niazi and coworkers, who evaluated the effectiveness of 1% trypsin and 1% proteinase K, with or without ultrasonic activation, on a multi-species biofilm. Trypsin with ultrasonic activation was able to effectively kill both aerobic and anaerobic bacteria and has the capability of disrupting the biofilm [126].

Agents that interfere with the cell wall, such as d-amino acids, specifically d-leucine has been demonstrated to bring about efficient dispersal of *Enterococcus fecalis* biofilms. It has been suggested that the dispersal of biofilms by sub-toxic concentrations of this agent reduces the success of resistant organisms [84]. Table 1 summarizes of the role of each category of agents in root canal disinfection.

#### 5.1.8. Intracanal Medicaments

Intracanal medicaments have conventionally been recommended as root canal dressing between appointments. However, with the increasing trend towards single visit endodontics, and the lack of a clear advantage of multiple visit treatments over single visit [127,128,129], the role of inter-appointment dressings is questionable and may wane with time. Nevertheless, considering the present evidence base (or lack of, thereof) to support one approach over the other, this section will briefly focus on the role of intracanal medicaments in root canal disinfection. The reader is referred to other reviews on this topic for details on the composition and mechanisms [130,131]. A systematic review on the effect of intracanal medicaments on bacterial biofilm concluded that these agents had limited action against a biofilm [132]. Furthermore, even if these agents are able to bring about bacterial killing inside a biofilm, it is unknown if they can disrupt or remove biofilms from the root canal system.

Calcium hydroxide has been shown to be ineffective against biofilms of *E. fecalis* even after 24 hours of treatment [133]. It was also reported that the addition of efflux pump inhibitors were unable to potentiate the antibiofilm activity of calcium hydroxide and nanoparticles of chitosan [133]. While this lack of effectiveness of calcium hydroxide was also true for multi-species biofilms, addition chitosan nanoparticles to calcium hydroxide appears to enhance the bacterial killing in a multi-species model over a 7 and 14 day period [134]. Nevertheless, it is unknown if this strategy per se can remove biofilms from within the radicular space. Furthermore, research is lacking in terms of the ability of intracanal medicaments to penetrate the EPS matrix of biofilms.

Antibiotic combinations have been studied over the past few years as a regimen during regenerative endodontic strategies. The literature is inconsistent on the effectiveness of double and triple antibiotic pastes (DAP and TAP) respectively against mono- and multi-species biofilms. It has been shown that TAP is significantly better than calcium hydroxide and chlorhexidine in disrupting biofilms of *E. fecalis* [6]. It has been suggested that 1 mg/mL DAP is needed to demonstrate any significant antibiofilm activity [135]. In addition, polymer nanofibers with TAP has been shown to bring about significant bacterial killing in a dual-species model composed of *Actinomyces naeslundii* and *E. fecalis* [136]. However, the possibility of root canal bacteria to develop resistance to antibiotics and possible allergic reactions in patients, remain important concerns with this category of medicaments. With the current available evidence, it remains unclear if intracanal medicaments are effective against multi-species biofilms.

### 5.2. Irrigant Activation Mechanisms

Complexity of the root canal anatomy and tenacious nature of the biofilms dictate that simple delivery of antimicrobial agents is not sufficient for disinfection of root canal systems. The focus is on developing methods that will satisfactorily deliver these antimicrobial agents into the complex anatomy, interfere with the adhesive mechanisms by inducing shear stress and disrupt the biofilms. 

#### 5.2.1. Sonics and Ultrasonics

Ultrasonic agitation can cause dis-agglomeration of the bacterial biofilm, thus re-suspending the bacteria in planktonic form which are then, more susceptible to antimicrobial irrigants. Also, any cavitation that may be produced, would cause temporary weakening of the cell membrane, thereby increasing the bacterial cell permeability to antimicrobial irrigants [137,138,139].

There is wide variability in the endodontic literature with regards to effectiveness of sonic and ultrasonics in removal of smear layer as well as antibacterial activity. In part, this may be due to differences in the study design, with the parameters for using these activation methods being inconsistent. Notable differences concerned include the volume of irrigating solution used, concentration, activation cycle and replenishment cycle of the solution, which result in inconclusive results [140,141]. From a logical standpoint, agitation of irrigating agents with sonic or ultrasonic should result in shear stresses that may cause detachment of the biofilms from the root canal walls [140]. It will also enable better penetration of irrigating agents into the lateral channels of the root canal system allowing better disinfection. However, there appears to be no strong evidence to demonstrate the clinical effectiveness of this approach.

#### 5.2.2. Light: Non-Coherent (Photoactivated Disinfection) and Coherent (Laser Activated Disinfection)

Since the concept of photoactivated disinfection against root canal biofilms has been discussed already, this section will only focus on the role of lasers against biofilms within root canals. Incomplete dissolution of intracanal biofilm seems to be a common finding after chemo-mechanical preparation of the root canal system [142,143]. Lasers that have a wavelength interacting with water molecules have been used to produce cavitation in liquids. When laser irradiation pulses, the cavitation effect produces a shockwave that can move the irrigating solution within the canal. One brand of Erbium:YAG (Er:YAG) laser propose its use in combination with a special tip to achieve the so-called Photon-induced photoacoustic streaming (PIPS) or irrigant in the canal. This device has been researched for removing debris and smear layer from the root canal system and the results seem positive [144].

There are only a few studies that evaluated laser activation of irrigants using a bioflm model; one of them examined the cleaning of biofilm-infected dentin on a bovine root canal comparing it with sonic or ultrasonic activation and needle irrigation. The authors showed favorable results for PIPS when compared to the other irrigant agitation methods [77]. Neelakantan et al., demonstrated that both diode and Er:YAG lasers were more effective than ultrasonic activation or syringe irrigation method for removing *E. fecalis* biofilms. However, this study reported no significant difference between Er:YAG and diode laser when a new irrigating agent (sodium hypochlorite mixed with etidronic acid) was used [81].

#### 5.2.3. Microbubble Emulsion

Halford et al., were the first to employ a microbubble emulsion to enhance the effect of sonic and ultrasonic agitation of sodium hypochlorite [145]. Essentially, the technique employs unstable gas-filled microbubbles that expand when exposed to ultrasonic waves. The dynamics thereby induced in the fluid would help in detaching surface adherent bacteria or biofilm destruction. In addition, it may also generate reactive oxygen species to exhibit an antibacterial effect. Microbubble emulsion in combination with ultrasonic agitation was shown to be superior than with sonic agitation [145]. This approach appears clinically interesting and warrants further research.

## 6. Future Directions

Despite the increasing knowledge of the microbial status of root canal systems, much still remains unknown. The reported success rates of root canal treatment have not undergone significant improvement [146,147,148,149]. From the clinical perspective, it is important to understand the aetiopathogenesis of periradicular periodontitis as a disease caused by microbial infection of the root canal system. Even though we know that root canal biofilms are complex, the literature unfortunately does not seem to offer due credence to understanding the dynamics between the components of a biofilm. Crosstalk between bacteria is a paradigm that has not be sufficiently studied thus far in the context of endodontic disease.

The authors of this paper call into action, the need for better understanding of the interactions between microbes in biofilms and how each organism influences the other. This, coupled with targeted therapeutic strategies may help improve the success rates of root canal treatment. Such strategies must focus on a step-wise approach from mono- to multispecies biofilms so as to develop a sufficient knowledge base on their mechanisms at a cellular level.

## 7. Conclusions

The root canal biofilm is a very complex, organized entity and it is difficult, but not impossible to duplicate its characteristics in in vitro experiments. Within root canal systems, the complexity is not only related to the nature of the biofilm, but also the complex anatomy, which houses tissue along with biofilms and removal of such biomasses is as relevant as being able to kill bacteria in biofilms.

Studies on monospecies or dual species biofilms may over-simplify this ecological phenomenon and may not be a true reflection of the results achievable in the clinical scenario.

The authors recommend that future studies should offer due credence to the complexity of the microbial biofilm and evaluate models to re-evaluate removal of the biomass from the root canals in addition to evaluating the action of novel antimicrobial agents on complex biofilms.

## Figures and Tables

**Table 1:** Summary of effect of commonly used root canal disinfectants on bacterial suspensions or biofilms in an endodontic disinfection model.

Antimicrobial Agent	Chemical Type	Concentration Used/Recommended in Root Canal Disinfection	Activity on Bacterial Suspensions (Root Canal Models Only)	ACTIVITY on Mono-Species or Multi-Species Biofilms (Endodontic Taxa Only)
Sodium hypochlorite (NaOCl)	Halogen releasing agent	1–6%	Yes	Yes
Chlorhexidine (CHX)	Bisbiguanide	2%	Yes	Unclear
Alexidine (ALX)	Bisbiguanide	1–2%	Yes	Unclear
Octenidine (OCT)	Bisbiguanide		Yes	Unclear
Iodine Potassium Iodide (IKI)	Halogen releasing agent	2–5%	Yes	Insufficient evidence
Ethylene diamine tetraacetic acid (EDTA)	Polyprotic acid	15–17%	No	No
Maleic acid	Diprotic acid	7%	Yes	Insufficient evidence
Peracetic acid	Organic peroxide	2.25%	Yes	Yes
MTAD	Mixture of antibiotic, organic acid (citric acid), detergent		Yes	Unclear
QMix	Mixture of CHX and EDTA		Yes	Yes
Etidronic acid (with 6% NaOCl)	Bis-phosphonate	18%	Yes	Yes
Curcumin	Phyto-polylphenol	—	Yes	Yes
Chitosan with Rose Bengal	Polysaccharide with photosensitiser		Yes	Yes
Silver nanoparticles	Metallic nanoparticle		Yes	Yes
Trypsin and Proteinase K	Enzymes	1%	Yes	Yes (Trypsin)
d-leucine	Amino acid		Yes	Yes

The term “unclear” has been used when methods other than confocal laser scanning microscopy have been used to detect the effect on biofilms.
